# Drug efficacy and toxicity prediction: an innovative application of transcriptomic data

**DOI:** 10.1007/s10565-020-09552-2

**Published:** 2020-08-11

**Authors:** Xuhua Xia

**Affiliations:** 1 Department of Biology, Faculty of Science, University of Ottawa, Ottawa, Ontario K1N 6N5 Canada; 2 Ottawa Institute of Systems Biology, Ottawa, K1H 8M5 Canada

**Keywords:** Transcriptomic efficacy, Transcriptomic toxicity, Toxicity prediction, Cystic fibrosis, Acute myeloid leukemia, Transcriptome, Drug development

## Abstract

Drug toxicity and efficacy are difficult to predict partly because they are both poorly defined, which I aim to remedy here from a transcriptomic perspective. There are two major categories of drugs: (1) restorative drugs aiming to restore an abnormal cell, tissue, or organ to normal function (e.g., restoring normal membrane function of epithelial cells in cystic fibrosis), and (2) disruptive drugs aiming to kill pathogens or malignant cells. These two types of drugs require different definition of efficacy and toxicity. I outlined rationales for defining transcriptomic efficacy and toxicity and illustrated numerically their application with two sets of transcriptomic data, one for restorative drugs (treating cystic fibrosis with lumacaftor/ivacaftor aiming to restore the cellular function of epithelial cells) and the other for disruptive drugs (treating acute myeloid leukemia with prexasertib). The conceptual framework presented will help and sensitize researchers to collect data required for determining drug toxicity.

**Electronic supplementary material:**

The online version of this article (10.1007/s10565-020-09552-2) contains supplementary material, which is available to authorized users.

## Introduction

The most desirable drug is of high efficacy, low toxicity (side effects), low chance of drug resistance, low cost, and low deleterious effect on the environment, e.g., no re-activation by bacterial species after human use (Xia 2017). Among these five key features, drug toxicity is perhaps the most difficult to define, quantify, and predict (Sosnin et al. 2019). In this review, I aim to introduce a standard definition for drug efficacy and drug toxicity from a transcriptomic perspective to facilitate their prediction in drug discovery in a transcriptomic context.

Drugs can be classified broadly into restorative and disruptive drugs. Restorative drugs aim to restore cellular functions. For example, in cystic fibrosis (CF) patients homozygous for the ΔF508 mutation (deletion of a phenylalanine at site 508) in the *CFTR* gene, the misfolded protein in endoplasmic reticulum (ER) is mostly degraded after failing to go through the quality control system (Fraser-Pitt and O'Neil 2015). The few CFTR proteins that do escape the degradation and are exported to their membrane location typically do not function very well. Thus, any modulators that can increase the export of CFTR protein to the membrane and improve its ion channel function would contribute to restoring the epithelial cell function and alleviate the associated symptoms (Deeks 2016; Sala and Jain 2018; Gentzsch and Mall 2018). Lumacaftor/ivacaftor for treating CF patients who are ΔF508 homozygotes is a drug combination representative of restorative drugs. From a transcriptomic perspective, the efficacy of such drugs is measured by how much they can reduce the difference in transcriptomic profile between patients and healthy controls, especially for a subset of genes directly related to the disease (Failli et al. 2019; Karagianni et al. 2019). Their toxicity is measured by the drug-induced differences in transcriptomic profile for genes that are not intended to be affected by the drug.

In contrast to restorative drugs, disruptive drugs are intended to disrupt cell growth and proliferation and to induce apoptosis. These drugs are used in the fight against pathogens or malignant cells (Moffat et al. 2014; Shoemaker 2006) without deleterious effect on normal human cells. Drug efficacy of disruptive drugs can be directly measured by the proportion of cancer cells or pathogens killed, from which one can obtain an estimate of the propensity of cancer cell or pathogen mortality. From a transcriptomic perspective, drug efficacy can be defined as an index of disruption, measured by the drug-induced difference in transcriptomic profile of malignant cells before and after drug use, especially the induction of apoptosis genes and activation of apoptosis pathways. The drug toxicity could be conceptually defined as drug-induced transcriptomic differences of normal cells before and after drug administration. In practice, this definition has limitations and alternatives are discussed.

I detail the definitions below, outline the rationale behind such definitions, and illustrate their applications that lead to meaningful quantification of drug efficacy and toxicity from transcriptomic data. Two sets of large-scale transcriptomic data are used for the illustration and can be downloaded from NCBI. I also include two supplemental files containing the data used in this paper, with detailed instructions on how to replicate the results in the paper. The first data set involves a restorative drug, i.e., lumacaftor/ivacaftor used to restore the cellular function of epithelial cells of CF patients with the double ΔF508 mutation (Kopp et al. 2019; Kopp et al. 2020). The second data set resulted from treating acute myeloid leukemia with prexasertib (Kaufmann and Li 2019).

## Drug efficacy and toxicity: restorative drug

Lumacaftor/ivacaftor for CF is a drug combination representative of restorative drugs. The majority of CF is caused by the deletion of F508 (ΔF508) in both alleles of the CFTR gene (Brockman et al. 2017; Esposito et al. 2016; Faure et al. 2016). The ΔF508 CFTR proteins cannot be folded properly in ER lumen and are mostly degraded after failing to be exported to the cell membrane to perform its ion channel function (Fraser-Pitt and O'Neil 2015). CFTR-Associated Ligand (CAL), an ER-localized protein, binds to ΔF508 CFTR, leading to degradation in the 26S proteasome (Bergbower et al. 2018) through the ubiquitin-proteasome pathway (Sondo et al. 2018). The few ΔF508 CFTR that do find their way to cell membrane do not function well due to severe gating defects (Bose et al. 2019). Thus, drugs that can decrease the degradation of ΔF508 CFTR protein, increase the export of ΔF508 CFTR protein to the plasma membrane, and improve its ion channel function, would contribute to restoring the epithelial cell function and alleviating the associated symptoms of cystic fibrosis. Such drugs and drug candidates include lumacaftor/ivacaftor (Deeks 2016; Gentzsch and Mall 2018; Kmit et al. 1865), tezacaftor/ivacaftor (Sala and Jain 2018; Faure et al. 2016; Donaldson et al. 2018), fatty acid cysteamine (Vu et al. 2017), or even rattlesnake phospholipase A2 (Faure et al. 2016). How to evaluate efficacy and toxicity of these drugs with transcriptomic data?

A recent study (Geo DataSets accession GSE124548) characterized whole-blood transcriptomic responses to lumacaftor/ivacaftor therapy in CF patients homozygous for ΔF508 (Kopp et al. 2019; Kopp et al. 2020). It gathered transcriptomic data for a total of 15,570 RNA and protein-coding genes from 20 CF patients before and after administration of lumacaftor/ivacaftor, as well as 20 non-CF individuals as control. Thus, the complete set of gene expression data is a 15,700 × 60 matrix. For each gene, there are 20 gene expression values for patients before the drug administration, 20 values for the same set of patients after the drug administration, and 20 values for healthy controls. I normalized the total number of read counts (i.e., the summation of each column of 15,570 values) to one million to facilitate comparison.

One might question the relevance of whole-blood transcriptomic data to CF drug efficacy. The most direct measure of efficacy would seem to be peeling off a piece of epithelium (especially those lining the airways) to test for the presence (or increased amount) of functional CFTR protein. If this invasive approach is not acceptable, then there are simple alternatives such as the conventional sweat test for efficacy. A reduction in the amount of chloride in sweat would seem to be an excellent index of efficacy for any CF drug. However, while lumacaftor/ivacaftor treatment does not seem to reduce chloride in sweat, the treated patients did report an improved quality of life.

Experimental evidence that implicated leucocytes in CF development has accumulated in the last 20 years. CF is made much worse by human immune responses mediated by leucocytes (mainly through neutrophils) (Makam et al. 2009; Tirouvanziam et al. 2006; Tirouvanziam et al. 2000; Tirouvanziam et al. 2008). Not only can a subset of neutrophils cause CF in mice, such neutrophils from CF patients can even transfer CF to mice (Genschmer et al. 2019). While it is not clear which subset of genes exhibit abnormal expression that leads to the full-fledged development of CF, it is clear that gene expression in leucocytes plays a key role in the development of CF (Lin et al. 2008). In addition to mRNA differences, microRNA miR-155 is also highly expressed in circulating CF neutrophils biopsied from CF patients (Bhattacharyya et al. 2011). For this reason, it is not outlandish to use whole-blood transcriptomic differences to characterize efficacy and toxicity of CF drugs.

Designate transcriptomes for patients before and after the administration of the drug as *P*_*b*_ and *P*_*a*_ (where subscripted *b* and *a* stand for before and after) respectively, and those for healthy controls as H (for healthy). Ideally one should compile a list of target genes that are particularly relevant to CF and formulate transcriptomic efficacy and toxicity based on how the drug treatment would restore their gene expression to that of healthy controls. However, given the existing knowledge on CF, there is practical difficulty in compiling such a set of target genes, so we will use all genes with expression levels clearly above background. Gene expression differs dramatically among genes, with S100A9 and EEF1A1 having mean expression equal to 10,611.94 and 8871.31, respectively, but many others with small values. I excluded genes with mean expression values lower than 10. This leaves 8558 genes.

The first 20 genes that differ most between H and P_b_ are listed in Table 1, together with differences between H and P_a_ and the results of significance tests. The first gene is *ARID3A* which belongs to the Arid (AT-rich interaction domain) family of DNA-binding proteins. At least one member of the family (*ARID3B*) is highly expressed in adult fibrotic lung tissue (Lin et al. 2008). *ARID3A* is expressed only in human B cells, but its function is little known (Nixon et al. 2004). The second gene (Table 1) is *STX3* which encodes a protein targeted to the apical membrane of epithelial cells and is crucial for the normal function of CFTR (Tang et al. 2011). The third gene is *SOD1* belonging to the superoxide dismutase family. Superoxide dismutases, especially extracellular ones, play a key role in preventing pulmonary fibrosis (Gao et al. 2008). These suggest that whole-blood transcriptomic data may shed light on mechanism of CF development.

**Table 1:** The first 20 genes that differ most in transcriptome between the control (H) and CF patients before drug administration (P_b_), together with associated *t* tests

Gene ID	MeanH^(1)^	MeanP_b_^(1)^	MeanP_a_^(1)^	t_H~Pb_^(2)^	p_H~Pb_^(2)^	t_H~Pa_	p_H~Pa_
*ARID3A*	63.5434	95.4289	82.7131	7.6575	3.2288E-09	3.8293	4.6701E-04
*STX3*	130.1254	202.3724	173.3726	7.5091	5.0883E-09	3.2182	2.6391E-03
*SOD1*	78.4708	55.6722	67.6733	7.3851	7.4545E-09	2.4629	1.8425E-02
*FUZ*	13.7661	10.1532	11.1206	7.3513	8.2743E-09	3.7607	5.7037E-04
*TRIM25*	121.4586	221.7997	174.1186	7.1480	1.5523E-08	2.8273	7.4488E-03
*MAPKAPK2*	86.6486	111.9230	106.6062	7.1071	1.7623E-08	5.1495	8.3141E-06
*FOSL2*	78.9880	151.0020	117.1378	6.8847	3.5231E-08	2.7905	8.1844E-03
*TMEM185B*	28.0728	41.2852	35.2257	6.7928	4.6956E-08	2.9412	5.5406E-03
*AP5B1*	88.5422	145.4842	119.1952	6.7822	4.8546E-08	3.1719	2.9942E-03
*CREBL2*	111.8329	84.8495	96.9893	6.7771	4.9325E-08	2.6558	1.1500E-02
*FAR1*	175.8316	233.5993	226.7157	6.7172	5.9497E-08	4.4820	6.5967E-05
*SRSF8*	102.1043	68.8498	76.8809	6.5682	9.4948E-08	4.4411	7.4762E-05
*ATG14*	43.0031	31.5710	34.6214	6.5661	9.5600E-08	4.3494	9.8853E-05
*ITGAM*	154.2052	260.6106	233.4123	6.5658	9.5689E-08	3.8977	3.8204E-04
*PPOX*	17.8127	12.5082	14.1883	6.5191	1.1081E-07	3.9620	3.1604E-04
*C16orf72*	275.0084	389.0771	344.2402	6.4950	1.1954E-07	3.2590	2.3602E-03
*MXD1*	602.1311	983.0411	820.7273	6.4777	1.2622E-07	2.9392	5.5701E-03
*MCTP1*	75.9118	103.7555	96.3500	6.4688	1.2977E-07	4.3876	8.8009E-05
*THAP11*	39.0977	30.2926	36.0012	6.4284	1.4738E-07	1.6531	1.0655E-01
*CARD6*	16.8386	26.6380	24.4786	6.4217	1.5052E-07	4.7320	3.0554E-05

^(1)^MeanH, MeanP_b_, MeanP_a_: mean expression for healthy control (H), CF patients before treatment and CF patients after treatment, respectively

^(2)^t_H~Pb_, p_H~Pb_: t and p values from *t* test between healthy control (H) and CF patients before treatment

^(3)^t_H~Pa_, p_H~Pa_: t and *p* values from *t* test between healthy control (H) and CF patients after treatment

It is almost never easy to identify key genes responsible for a disease. The patient group may exhibit altered expression of many genes including the disease-causing genes and those representing secondary responses. The healthy controls may include individuals who are about to have the disease and exhibit disease-specific gene expression patterns but have not yet manifested the disease symptoms. In this context, it is encouraging to identify genes such as *ARID3A*, *STX3*, and *SOD1* that are known to be directly or indirectly related to CF.

Drug efficacy is the summation of everything better after drug treatment than before drug treatment. *ARID3A* expression is much higher in CF patients than in healthy control (95.4289 vs 63.5434, Table 1). After drug treatment, *ARID3A* expression is reduced to 82.7131 (Table 1), closer to that of the healthy control by 12.7158. Designate mean expression of H, P_b_, and P_a_ as MeanH, MeanP_b_ and MeanP_a_, respectively. Now for *ARID3A*

1 $$ {D}_{\mathrm{MeanH}\sim \mathrm{Mean}{\mathrm{P}}_{\mathrm{b}}}=\left|\mathrm{MeanH}-\mathrm{Mean}{\mathrm{P}}_{\mathrm{b}}\right|=\left|63.5434-95.4289\right|=31.8855 $$

2 $$ {D}_{\mathrm{MeanH}\sim \mathrm{Mean}{\mathrm{P}}_{\mathrm{a}}}=\left|\mathrm{MeanH}-\mathrm{Mean}{\mathrm{P}}_{\mathrm{a}}\right|=\left|63.5434-82.7131\right|=19.1697 $$

3 $$ {\Delta}_D={D}_{\mathrm{MeanH}\sim \mathrm{Mean}{\mathrm{P}}_{\mathrm{b}}}-{D}_{\mathrm{MeanH}\sim \mathrm{Mean}{\mathrm{P}}_{\mathrm{a}}}=31.8855-19.1697=12.7158 $$

where Δ_*D*_ is desirable if positive and undesirable if negative. A better replacement of ${D}_{\mathrm{MeanH}\sim \mathrm{Mean}{\mathrm{P}}_{\mathrm{a}}}$ and ${D}_{\mathrm{MeanH}\sim \mathrm{Mean}{\mathrm{P}}_{\mathrm{b}}}$ is the t statistic which incorporates the standard error (SE) of the differences. Again for *ARID3A,*

4 $$ {t}_{\mathrm{MeanH}\sim \mathrm{Mean}{\mathrm{P}}_{\mathrm{b}}}=\frac{\left|\mathrm{MeanH}-\mathrm{Mean}{\mathrm{P}}_{\mathrm{b}}\right|}{\mathrm{SE}}=\frac{\left|63.5434-95.4289\right|}{4.1640}=7.6575 $$

5 $$ {t}_{\mathrm{MeanH}\sim \mathrm{Mean}{\mathrm{P}}_{\mathrm{a}}}=\frac{\left|\mathrm{MeanH}-\mathrm{Mean}{\mathrm{P}}_{\mathrm{a}}\right|}{\mathrm{SE}}=\frac{\left|63.5434-82.7131\right|}{5.0061}=3.8293 $$

6 $$ {\Delta}_t={t}_{\mathrm{MeanH}\sim \mathrm{Mean}{\mathrm{P}}_{\mathrm{b}}}-{t}_{\mathrm{MeanH}\sim \mathrm{Mean}{\mathrm{P}}_{\mathrm{a}}}=7.6575-3.8293=3.8282 $$

**Fig. 1:**
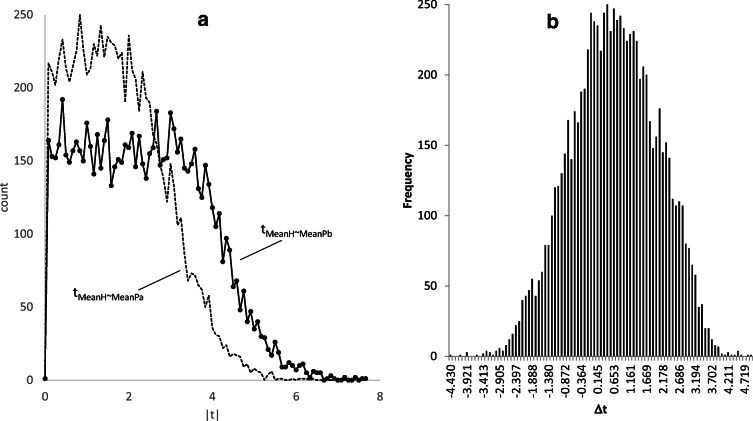
Distribution of two sets of t values designated t_MeanH~MeanPb_ and t_MeanH~MeanPa_ (**a**) and distribution of Δt (**b**). t_MeanH~MeanPb_ and t_MeanH~MeanPa_ measure deviation of gene expression of CF patients from that of healthy controls (H) before and after the drug treatment, respectively. Shifting of t_MeanH~MeanPa_ towards smaller values relative to t_MeanH~MeanPb_ indicates positive drug effect. Large Δt values indicate desirable drug effect

${t}_{\mathrm{MeanH}\sim \mathrm{Mean}{\mathrm{P}}_{\mathrm{b}}}$ and ${t}_{\mathrm{MeanH}\sim \mathrm{Mean}{\mathrm{P}}_{\mathrm{a}}}$ values measure deviation of gene expression in CF patients from that of healthy controls before and after drug treatment, respectively. Ideally, all ${t}_{\mathrm{MeanH}\sim \mathrm{Mean}{\mathrm{P}}_{\mathrm{a}}}$ values would be zero; i.e., the gene expression is perfectly restored to that of healthy controls. In the case of *ARID3A*, although ${t}_{\mathrm{MeanH}\sim \mathrm{Mean}{\mathrm{P}}_{\mathrm{a}}}$ is not zero, it is at least smaller than ${t}_{\mathrm{MeanH}\sim \mathrm{Mean}{\mathrm{P}}_{\mathrm{b}}}$; i.e., the gene expression is closer to that of the healthy control after drug treatment than before drug treatment. The distribution of ${t}_{\mathrm{MeanH}\sim \mathrm{Mean}{\mathrm{P}}_{\mathrm{b}}}$ and ${t}_{M\mathrm{eanH}\sim \mathrm{Mean}{\mathrm{P}}_{\mathrm{a}}}$ for the 8558 genes (Fig. 1a) suggests positive drug effect. That is, the distribution of ${t}_{\mathrm{MeanH}\sim \mathrm{Mean}{\mathrm{P}}_{\mathrm{a}}}$ has shifted towards smaller values relative to the distribution of ${t}_{\mathrm{MeanH}\sim \mathrm{Mean}{\mathrm{P}}_{\mathrm{b}}}$.

Δt in Eq. (6) measures drug effect on a specific gene (*ARID3A*). If the drug is efficacious, we expect most genes to have positive Δt values than genes with negative Δt values. Among the 8558 genes, 5710 has positive values and 2848 have negative values. The distribution of the 8558 Δt values (Fig. 1b) suggests a mean Δt greater than 0. The 20 genes with the most negative Δ_*t*_ values, i.e., genes with the greatest side effect (or toxicity effect), are listed in Table 2.

**Table 2:** The first 20 genes that have the most negative Δt values (negative Δt means gene expression deviating even more from healthy control after drug treatment and is therefore undesirable). Column headings same as in Table 1

ID	MeanH	MeanPb	MeanPa	t_H~Pb_	t_H~Pa_	Δt
C1orf132	18.68247	15.5787	12.03481	1.720729	6.150399	− 4.42967
TTN	81.53304	81.38701	43.38747	0.00516	4.167737	− 4.16258
MFSD4B	16.52881	14.63438	12.18393	1.095759	5.10132	− 4.00556
STK39	21.85199	21.75404	17.98743	0.039667	4.031563	− 3.9919
PPCDC	30.06767	28.48239	23.52954	0.490643	4.453355	− 3.96271
RC3H2	83.41912	76.31473	71.25956	1.995434	5.645152	− 3.64972
EZH1	91.2039	85.87195	75.65626	1.542998	5.042697	− 3.4997
HELLS	14.71387	12.28021	10.44332	1.345294	4.786592	− 3.4413
ANKRD36B	18.44703	13.77633	9.970375	1.660923	5.047357	− 3.38643
LINC01138	12.93422	11.9653	9.190063	0.536082	3.917247	− 3.38116
CNNM3	19.83114	19.08763	16.34129	0.294289	3.667048	− 3.37276
ALG13	26.53309	26.12448	21.08266	0.099649	3.45059	− 3.35094
PLA2G6	13.24867	11.03068	9.140276	1.493657	4.754572	− 3.26091
ANKRD36	41.56848	34.32497	29.99873	1.730452	4.990922	− 3.26047
ANKRD36C	28.59038	25.00426	20.63043	0.863642	4.101587	− 3.23794
CCNH	46.21754	45.87676	39.76174	0.100098	3.291842	− 3.19174
LOC105371224	17.35159	16.08431	10.64258	0.405861	3.488259	− 3.0824
ABCA5	25.68626	25.31876	21.00044	0.15661	3.216822	− 3.06021
DYRK1A	279.4142	273.7788	251.3844	0.851491	3.878661	− 3.02717
PIGG	33.24765	31.67206	26.59577	0.390097	3.411989	− 3.02189

I now define an index of drug desirability as

7 $$ {I}_{DD}=\frac{\sum_{i=1}^{N_{\mathrm{gene}}}{\Delta}_{t\cdotp i}}{N_{\mathrm{gene}}}=\frac{\sum_{i=1}^{8558}{\Delta}_{t\cdotp i}}{8558}=0.6093 $$

where *I*_*DD*_ is simply an average of Δ_*t*_. The drug is desirable if *I*_*DD*_ > 0, undesirable if *I*_*DD*_ < 0, and neither desirable nor undesirable if *I*_*DD*_ = 0 (the null hypothesis). For the CF transcriptomic data, *I*_*DD*_ is highly significantly greater than 0 based on the 8558 Δ_*t*_ values (*t* = 41.8478, *DF* = 8557, *p* < 10^−20^). The standard error (SE) of Δ_*t*_ is 0.01456, so that 95% confidence interval of *I*_*DD*_ is (0.58073, 0.63781).

Given 5710 genes with positive Δ_*t*_ and 2848 genes with negative Δ_*t*_, the drug efficacy and drug toxicity can be defined as

8 $$ E=\frac{\sum_{i=1}^{5710}{\Delta}_t}{8558}=0.90402;\mathrm{for}\ {\Delta}_t>0 $$

9 $$ T=\frac{\sum_{i=1}^{2848}{\Delta}_t}{8558}=-0.29475;\mathrm{for}\ {\Delta}_t<0 $$

10 $$ {I}_{DD}=E+T $$

which implies that a drug can become more desirable by either increasing *E* or reducing *T*.

To generate a more informative *I*_*DD*_, one should use a fixed set of candidate genes known *a priori* to be relevant for the disease instead of using all 8558 genes. We can use this set of candidate genes to replace the 8558 genes in the computation. For disruptive drugs aiming to kill cancer cells, such a fixed set of genes could simply be all genes involved in apoptosis pathways. For restoration drugs aiming to restore a specific function, then all genes contributing to the function can be included in the set.

Suppose a researcher has done a similar experiment with a new drug, or the same drug with a different dose, and wish to compare his *I*_*DD*_, *E* and *T* against those reported above. He may compute *I*_*DD*_ from his experimental result with the same set of genes. If the lower limit of his 95% confidence interval for *I*_*DD*_ is greater than my calculated *I*_*DD*_ (= 0.6093) or if his *I*_*DD*_ is greater than the upper limit of my 95% confidence interval (= 0.63781), then he may conclude that his drug or his dosage is more desirable than the lumacaftor/ivacaftor treatment. He can then further dissect the result to see whether his increase in *I*_*DD*_ is due to increased *E* or reduced *T*.

## Drug efficacy and toxicity: disruptive drugs

Disruptive drugs aim to induce large changes in the target cells, ideally leading to cell death. Suppose we are treating liver cancer with a particular drug. We would need transcriptomes from normal liver cells and malignant liver cells before and after drug treatment, represented as *GE*_nb_, *GE*_na_, *GE*_mb_, and *GE*_ma_, where *GE* stands for gene expression, and the subscript n stands for normal, m for malignant, b for before, and a for after. I will first start with a general case with no specific set of target genes, and then narrow down to a set of genes involved in apoptosis.

### Drug efficacy with no specific set of target genes

For an anti-cancer drug, it is desirable to disrupt the cancer cell as much as possible, so *GE*_mb_, and *GE*_ma_ should differ as much as possible. For M genes with gene expression clearly above background, the transcriptomic efficacy (*E*) is defined as the mean |t| value:

11 $$ E=\frac{\sum_{i=1}^M\left|{t}_{i,{GE}_{\mathrm{ma}}\sim {GE}_{\mathrm{mb}}}\right|}{M} $$

Take for example the transcriptomic data (GSE131912) for treating acute myeloid leukemia with 10 nM prexasertib (Kaufmann and Li 2019), with three controls (CTRL) and three treatments (TREAT). I again normalized each of the six columns of data (3 CTRLs and 3 TREATs) to have a summed total of 1,000,000. After excluding genes with mean expression smaller than 10, I have 9446 genes remaining. The resulting *E* is

12 $$ E=\frac{\sum_{i=1}^{9446}\left|{t}_{i,{GE}_{\mathrm{ma}}\sim {GE}_{\mathrm{mb}}}\right|}{9446}=5.54566 $$

It is easy to test if this *E* = 5.54566 is statistically significant. The expected |t| value for any degree of freedom (*ν*) can be obtained by the following equation:

13 $$ E\left(t|\nu \right)=\frac{\int_0^{\infty }t\cdotp f\left(t|\nu \right) dt}{\int_0^{\infty }f\left(t|\nu \right) dt} $$

where *f*(*t*| *ν*) is the probability density function of t distribution given *ν*. In our case with *ν* = 4 (for a *t* test with 3 CTRLs and 3 TREATs), the expected |t| value is 1 when the null hypothesis of no difference is true. Therefore, we can do a simple *t* test as

14 $$ t=\frac{E-E\left(t|\nu \right)}{\mathrm{SE}}=\frac{5.54566-1}{0.06379}=71.26233 $$

where SE is the standard error of the 9446 t values used to calculate *E* in Eq. (12). The *p* value is effectively 0. In other words, the prexasertib treatment very strongly perturbed gene expression.

A multitude of diversifying lineages has been reported in tumors (Bailey et al. 2020; Turajlic et al. 2019; Wu et al. 2016), which can complicate transcriptomic data analysis (Xia 2017; Navin 2015). It would be interesting to know if a certain anti-cancer drug will perturb all different cancer cell lineages or just a subset of the lineages. An anti-cancer drug against one or only a subset of proliferating lineages will not be efficacious against the cancer.

### Drug efficacy with a set of target genes such as genes involved in apoptosis

Although one could calculate E by using all genes whose expressions are not too low, as is done above, the *E* value would be more informative if we use a set of candidate genes more relevant to the conventional sense of efficacy. For example, if the drugs are for inducing apoptosis in cancer cells, then we would be more interested in 80 or so apoptosis genes involved in extrinsic and intrinsic apoptosis pathways (Burke 2017), which we can obtain by using databases such as KEGG (Kanehisa 2002) or apoptosis database specific for human cancer such as ApoCanD (Kumar and Raghava 2016).

For illustration, I downloaded the 82 sequences for proteins involved in apoptosis from ApoCanD. From the same set of gene expression data on treating acute myeloid leukemia cells with prexasertib (Kaufmann and Li 2019) that I used in the previous section, there are 66 out of the 82 genes with mean gene expression greater than 10. The first 20 genes with the greatest difference between the control and the prexasertib treatment are listed in Table 3. In this case with a fixed set of genes, *E* is calculated as

15 $$ {E}_{\mathrm{apoptosis}}=\frac{\sum_{i=1}^{66}\left|{t}_{i,{GE}_{\mathrm{m}\cdotp \mathrm{a}}\sim {GE}_{\mathrm{m}\mathrm{b}}}\right|}{66}=7.12677 $$

**Table 3:** The first 20 genes that differ most in transcriptome between the three controls (CTRL) and three treatments (TREAT) with prexasertib, together with expression counts and associated *t* tests

Gene	CTRL1	CTRL2	CTRL3	TREAT1	TREAT2	TREAT3	T	p
TNFSF10	6.020	5.498	5.363	69.655	65.721	66.600	51.041	8.82E-07
CASP2	151.090	148.349	154.388	77.283	80.477	77.215	35.568	3.73E-06
CSE1L	268.935	257.947	259.631	120.659	109.006	129.532	20.777	3.17E-05
BBC3	9.302	14.353	15.471	188.474	161.038	186.963	18.210	5.35E-05
AK2	578.682	579.970	575.428	291.260	327.285	342.539	16.882	7.22E-05
CASP4	50.896	54.203	53.591	79.731	75.904	78.485	16.571	7.77E-05
ANP32A	462.079	493.671	465.000	202.516	238.210	233.937	16.462	7.97E-05
MYD88	108.917	93.516	84.762	328.306	300.993	292.179	16.306	8.28E-05
CASP10	17.243	24.593	23.021	59.258	56.823	60.428	15.044	0.000114
BCL2	108.373	86.368	98.274	9.789	9.782	12.872	13.482	0.000175
MAPK8	41.322	40.927	44.489	26.144	23.170	24.933	12.324	0.000249
BCL2L13	78.444	77.788	78.923	128.507	141.324	151.767	9.229	0.000766
HTRA2	15.373	16.117	16.741	6.802	9.382	9.010	8.560	0.001023
BAX	83.903	85.291	83.068	194.516	198.765	241.131	8.552	0.001026
XAF1	31.816	38.980	48.881	733.424	496.670	648.242	8.446	0.001077
CASP1	19.641	19.759	19.598	70.043	52.867	59.194	8.182	0.001215
MOAP1	19.148	18.843	22.192	29.958	29.029	28.812	8.181	0.001216
MCL1	342.193	341.233	337.354	1293.968	940.773	1097.231	7.539	0.001658
NAIP	6.241	7.171	7.744	30.464	33.804	23.205	6.998	0.002195
CASP8	38.346	34.719	32.370	54.060	48.777	53.710	6.996	0.002197

This *E*_apoptosis_ from the 66 genes involved in apoptosis is greater than *E* (= 5.54566) from all 9446 genes, suggesting that gene expression of apoptosis genes has been changed more by the drug than that of an average gene. One question that a researcher is interested in answering is whether this difference is statistically significant. I tested the difference between the 66 t values from apoptosis genes against the 9446 t values from the 9446 genes. The difference is significant, with *t* = 2.059644, *DF* = 9510, *p* = 0.03946. Thus, the drug altered expression of apoptosis genes more than it does to an average gene.

One may criticize the formulation of *E* in Eq. (11) as being only an index of gene expression disruption. If the drug is to induce apoptosis, then the efficiency should be defined as the propensity of tumor cell death (p). This *p* is typically dose-dependent for a drug. Table 4 illustrates such a set of fictitious data with measured dose-dependent cancer cell mortality (the first three columns), as well as expression for two apoptosis-related genes (*AG1* and *AG2*). As there are 80 or so genes closely involved in various apoptosis pathways, a real data set could include 80 or so columns in Table 4, each column representing dose-dependent expression of a gene. For simplicity we illustrate with only two genes.

**Table 4:** Sample data of dose-dependent cancer cell mortality and the expression of two genes related to apoptosis (AG1 and AG2)

Dose	N_Cell_^(1)^	N_Dead_^(2)^	*AG1*	*AG2*
0	3000	100	10	23
50	2400	130	1500	200
100	3100	350	2000	500
150	3000	500	2500	750
200	2000	600	2400	950
250	3000	1500	1600	1000
300	2600	1700	1500	1500
400	2800	2450	200	1900
500	2800	2600	100	2000

^(1)^N_Cell_: number of counted cancer cells

^(2)^N_Dead_: number of dead cancer cells

From the first three columns in Table 4, one can obtain the dose-dependent *p* using logistic regression (Berkson 1944), which would give us

16 $$ p=\frac{e^{a+b\cdotp \mathrm{Dose}}}{1+{e}^{e^{a+b\cdotp \mathrm{Dose}}}}=\frac{e^{-3.3827+0.0130\mathrm{Dose}}}{1+{e}^{-3.3827+0.0130\mathrm{Dose}}} $$

**Fig. 2:**
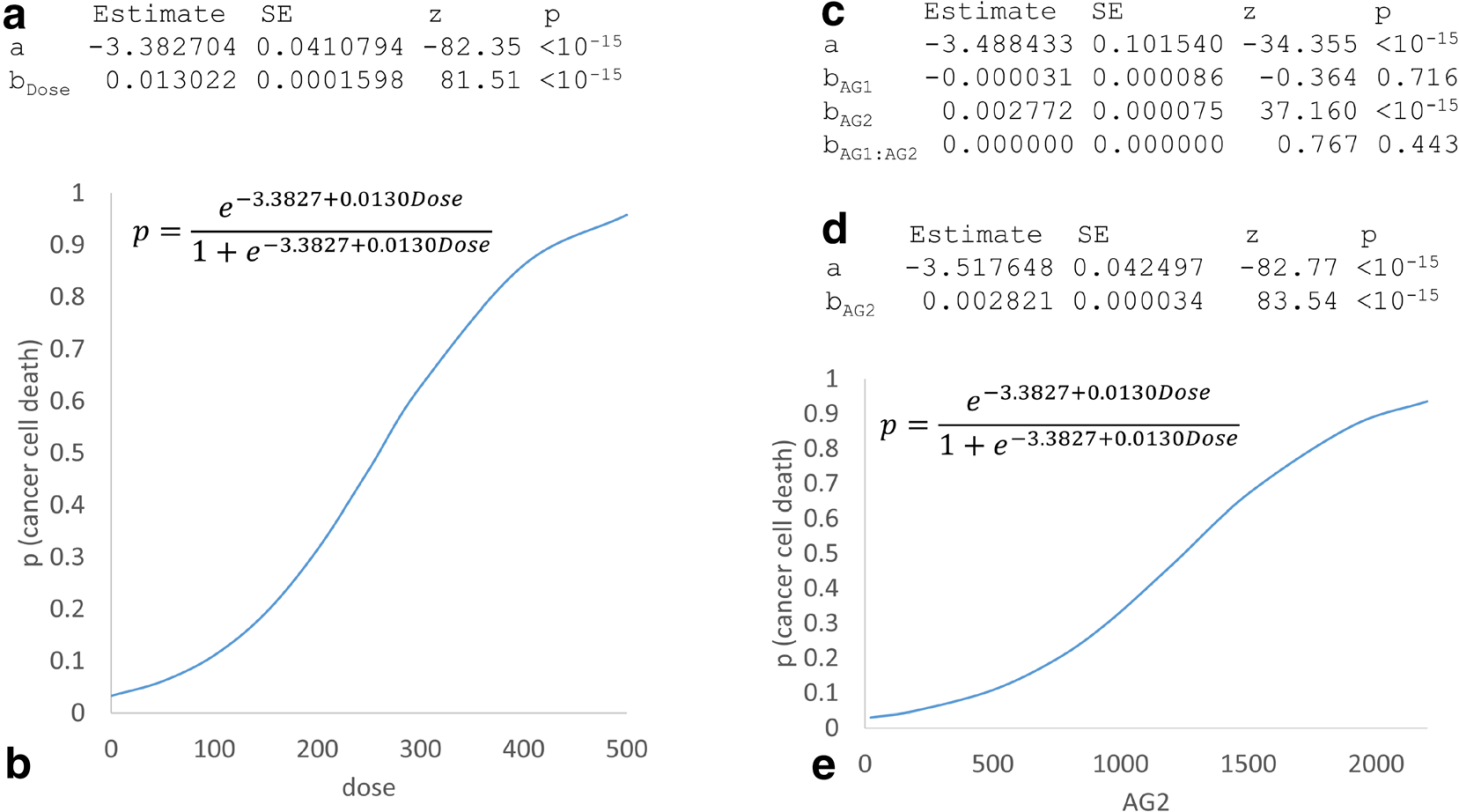
Results of analyzing data in Table 4 by logistic regression and generalized linear model. (**a**) Parameter estimation for a model of dose-dependent mortality (p) of cancer cells. (**b**) Visualization of the fitted model. (**c**) Parameter estimation for a model fitting cancer cell mortality on the expression of two genes (*AG1* and *AG2*), with *AG1* and the *AG1*:*AG2* interaction being not significant. (**d**, **e**) The best model with *p* plotted over gene expression of *AG2*

*p* is highly significantly related to dose (Fig. 2a) and the relationship is depicted in Fig. 2b. Because *p* seems to be a direct measure of the drug efficacy in killing cancer cells, what is the need for a transcription-based index of efficacy?

There are two arguments for the relevance of transcription data. First, we wish to know which gene whose altered expression may have contributed to the observed death of cancer cells. We can use generalized linear model (Nelder and Wedderburn 1972) to fit the relationship between *p* and *AG1* and *AG2*. As shown in Fig. 2c, *AG1* is not related to cancer cell mortality *p*, but *AG2* is highly significantly related to *p*. Also, there is no interaction between *AG1* and *AG2* (Fig. 2c). The best model, based on either likelihood ratio test or information-theoretic indices such as AIC or BIC (Burnham and Anderson 2002; Xia 2009), has * AG2* as the only independent variable. The fitted model (Fig. 2 d and e) shows the *AG2*-dependent drug efficacy. Such knowledge gains us a better understanding of the mechanistic basis of cancer cell death; i.e., the drug may have induced apoptosis through the pathway with a strong dependence on differential *AG2* expression. Second, alteration of gene expression typically occurs much earlier than cell death, so an efficacy index based on gene expression (especially those directly related to apoptosis) is likely more sensitive than one based on observed cell death.

Different drugs may target different sets of gene with totally different outcome in terms of transcriptome response although all of them may be highly efficient treatments against a certain disease. For example, some anti-cancer drugs aim to decrease expression of anti-apoptotic genes such as BCL-2, BCL-XL, and MCL1 (Luo et al. 2014; Sattler et al. 1997; Beroukhim et al. 2010), or increase the expression of pro-apoptotic genes such as BID, BIM, BAD, PUMA, and NOXA (Happo et al. 2010; Slinger et al. 2016; Zhang et al. 2013). Both types can lead to mitochondrial outer membrane permeabilization and subsequent activation of apoptosis agents such as caspases. If one compares transcriptomic efficacy of drugs suppressing the expression of anti-apoptotic genes such as BCL-2, BCL-XL, and MCL1 against transcription efficacy of drugs increasing pro-apoptotic genes such as BID, BIM, BAD, PUMA, and NOXA, then one would be comparing apples and oranges. In such cases, cancer cell mortality is a more general measure of drug efficacy. In short, the transcriptomic efficacy complements but does not replace the measure of cancer cell mortality as drug efficacy.

### Toxicity

A good drug should have high efficacy but low toxicity. For disruptive drugs, the transcriptomic toxicity is difficult to define except for the simplest cases such as skin cancer and some mouth cancer where (1) the tumor and the surrounding normal tissues are clearly distinguishable and (2) topical chemotherapy is used so that the tumor and the surrounding normal tissues are subject to the same treatment. In such cases, GE_m.b_ and GE_m.a_ can be characterized from the tumor, and GE_n.b_ and GE_n.a_ can be characterized from surrounding normal tissues. Transcriptomic toxicity T can then be calculated from GE_n.b_ and GE_n.a_,

17 $$ T={\sum}_{i=1}^M\left|{t}_{i,{GE}_{\mathrm{n}\cdotp \mathrm{a}}\sim {GE}_{\mathrm{n}\cdotp \mathrm{b}}}\right| $$

Again, the expected t, when there is no difference between *GE*_n.a_ and *GE*_n.b_, is specified in Eq. (13), which allows us to carry out a significance test of whether the drug has statistically significant transcriptomic toxicity.

E and T values should mainly be used to facilitate comparisons. If we have a new drug with an E value much greater than that for the old one, but a T value that is similar to, or smaller than, that for the old one, then we would be inclined to choose the new one over the old one. Similarly, if a heavier dose of prexasertib leads to much higher E but the same T, then the heavier dose is preferred.

The transcriptomic toxicity defined in Eq. (17) is limited for two reasons. First, GE_n.a_ typically cannot be measured because anti-cancer drugs almost invariably have strong side effect so it is consequently unethical to recruit heathy human subjects to take the drugs for measuring GE_na_. Prednisone (a glucocorticosteroid) used in some anti-cancer chemotherapies was the only one tested with healthy volunteers, but with only a single dose, causing a 72% decrease of the total lymphocyte number and a 97% decrease in total eosinophil count (Schuyler et al. 1984). Such a study would not be possible today. Second, when anti-cancer drugs are infused intravenously, numerous numbers of tissues and cell lineages are affected. A reasonable assessment of toxicity would need GE_nb_ and GE_na_ from all of these affected tissues. One alternative is to use animal models of human diseases such as mouse models of human cancer (Borowsky 2011; Cheon and Orsulic 2011; Rudin et al. 2019; Swiatnicki and Andrechek 2019), especially when oncogenes and tumor-suppressor genes can be conditionally turned on or off. These animal models allow us to measure GE_nb_ and GE_na_ as well as GE_mb_ and GE_ma_ for a variety of tissues. Another alternative is to use cell lines.

However, in spite of these two available alternatives (animal models and cell lines), transcriptomic cancer studies tend to measure only GE_mb_ and GE_ma_, but almost never GE_nb_ and GE_na_. It is too wasteful to collect transcriptomic data that cannot be used to quantify toxicity without which one cannot say whether one drug is more preferable than another. This general negligence to collect relevant data to estimate transcriptomic toxicity hinders cancer research and informed decision-making in drug administration. I hope that the definitions and illustrations I used in this paper will encourage researchers to collect more complete and informative data in the future and to formulate better indices of efficacy and toxicity.

## Conclusion

Drug toxicity prediction is a difficult subject, made more so by a lack of definitions. I have proposed informative definitions for transcriptomic efficacy and toxicity that are easy to use in real research settings with transcriptomic data. The conceptual framework associated with the definitions also highlights the general negligence of researchers in collecting data relevant to measure drug toxicity. I expect these definitions will result in significant improvement of accuracy and precision in drug development.

## Electronic supplementary material

CF_result.xlsx includes gene expression data for cystic fibrosis and results derived from the data. Prexsertib_result.xlsx includes gene expression data for acute myeloid leukemia treated with prexasertib, and results derived from the data. Each file contains a ReadMe sheet with details of data analysis.

ESM 1 (XLSX 20584 kb)

ESM 2 (XLSX 8443 kb)
